# Allopathic and traditional health practitioners’ collaboration

**DOI:** 10.4102/curationis.v38i2.1495

**Published:** 2015-07-23

**Authors:** Dalena van Rooyen, Blanche Pretorius, Nomazwi M. Tembani, Wilma ten Ham

**Affiliations:** 1 School of Clinical Care Sciences, Nelson Mandela Metropolitan University, South Africa; 2 Director Research Capacity Development, Nelson Mandela Metropolitan University, South Africa; 3 Director Professional Nurses, Eastern Cape, South Africa; 4 Department of Nursing, Nelson Mandela Metropolitan University, South Africa

## Abstract

**Background:**

Professional collaboration between traditional and allopathic health practitioners in South Africa is proposed in the *Traditional Health Practitioners Act* and could benefit and complement healthcare delivery.

**Objectives:**

To explore and describe the collaborative relationship between allopathic and traditional health practitioners regarding the legalisation of traditional healing, and these health practitioners’ views of their collaborative and professional relationship, as role-players in the healthcare delivery landscape in South Africa.

**Methods:**

A qualitative design was followed. The research population comprised 28 participants representing three groups: allopathic health practitioners (*n* = 10), traditional healers (*n* = 14), and traditional healers who are also allopathic health practitioners (*n* = 4). Purposive and snowball sampling was used. Data collection involved unstructured interviews, a focus group interview and modified participant observation.

**Results:**

Results indicate both allopathic and traditional health practitioners experienced negative attitudes towards each other. Mutual understanding (in the form of changing attitudes and communication) was considered crucial to effective collaboration between these two health systems. Participants made suggestions regarding capacity building.

**Conclusions:**

Considering realities of staff shortages and the disease burden in South Africa, facilitating collaboration between allopathic and traditional health practitioners is recommended. Recommendations could be used to develop strategies for facilitating professional collaboration between traditional and allopathic health practitioners in order to complement healthcare delivery.

## Introduction

Every society has various systems in place to maintain and restore well-being (Figueras & McKee 2012:5). These systems are influenced by differences between cultures and their understanding of health and disease. Additionally, these systems may include the alternative medical system, traditional healing, and the allopathic or professional health systems (McCleod & Chung 2012).

Members of rural communities, such as those living in the rural areas of the Eastern Cape, generally display similar help-seeking behavioural pattern. These may include firstly using the lay referral system of self-medication. This includes self-medication with patent medicines, traditional folk remedies and diet, followed by consultation with lay persons who are experienced regarding the health issue in question, and the the lay referral system (Abubakar *et al.* 2013:2). If there is no improvement, the sick person may consult a healer from either the allopathic health sector, or the traditional health sector (Abubakar *et al.* 2013:2).

Health seeking behaviour and the choice whether or not to consult an allopathic or traditional healer is a complex process and is determined by the chronicity/severity of the disease, attribution of causation of ill-health to supernatural sources, and as a preventative measure against possible ill-health (Moshabela 2012:26). However, these systems differ substantially from western biomedicine that approaches ill-health from the perspective of *what* caused it and *how*, whereas traditional healing deals with *who* caused it and *why* (Juma 2011:16). The allopathic health system includes physicians of all specialities as well as recognised allied medical disciplines, for example, nurses, physiotherapists, and radiotherapists and is located within a scientific paradigm, as it is characterised by the application of scientific medical knowledge and technology to health and the healing process (Kreitzer, Kligler & Meeker 2009:4).

The traditional health system, on the other hand, relies exclusively on observation and practical experience, handed down from generation to generation verbally or in writing (World Health Organization 2002:3). According to traditional African cosmology, the universe comprises two worlds; ‘the world in which man lives’, and ‘the world of the ancestral spirits’. As spiritual beings, the ancestral spirits are ‘invisible members of society who care for and carry responsibility for the actions of their descendants’. ‘Health, prosperity, and misfortune or ill-health are attributed to the continued goodwill or wrath of the ancestors by traditional communities’ (Ozumba 2004:3). Traditional healers in South Africa can include diverse categories such as the following:

- traditional practitioners (*Ixhwele*), who are predominantly men who specialise in the use of herbal medicines
- diviners (*Igqira*), who are predominantly women who are called by the ancestors to become a diviner to enable them to acquire the knowledge and skills of traditional healing (Jolles & Jolles 2000:230; Steinglass 2002:32)
- faith healers, who are people who use the power of suggestion, prayer, and faith in God to promote healing (Abubakar *et al.* 2013:2)
- and traditional birth attendants, or traditional midwives, who are middle-aged or elderly ladies with no formal training who attend to women during pregnancy, labour, and the post-natal period by using herbs to facilitate delivery or cause bleeding of the uterus post-nataly (Owens-Ibie 2011).

Globally, and in South Africa there is an increased interest and demand for the use of both traditional and allopathic health systems (Adams *et al.* 2009:793). For years there has been a growing pattern of using both health systems by persons moving from one sector of the healthcare system to the other, in search of diagnosis, healing or other services, or using both systems simoultaneaously (Adams *et al.* 2009:793; Frenkel *et al.* 2008:178; Torri 2012:34).

Professional collaboration between health systems, where both systems can complement each other, is, therefore, desirable and requires collaboration between the systems (Adams *et al.* 2009:793). The phenomenon of dual usage of medical resources is significant, especially for this study because it provides a basis for attempts at collaboration between modern and traditional healing (Torri 2012:34–35). This is not new to Pretorius (1991:11) who, in his analogical model of the Biomedical and Traditional Medical Relationship, advocates a new type of national healthcare delivery system. Traditional medicine can be made relevant through either an inclusive parallel system, whereby traditions other than allopathic medicine are recognised legally, thus, two or more systems of health co-exist, through integration of both systems (Pretorius 1991:11). When a relationship of complementarity and co-operation exists, traditional and modern medicine co-exist as two independent sectors, each acknowledging and considering the uniqueness of the other (Pretorius 1991:11).

The formal recognition of traditional healing, and its integration or incorporation into existing healthcare services, has been controversial for some time. Many arguments have been offered for and against their incorporation. Wiese and Oster (2010:416) as well as Abdullahi (2010:115) argue that part of the misunderstanding regarding African traditional healers emanates from a historical perspective. As Mulaudzi (2001:15) points out, missionaries were particularly negative towards traditional healers, viewing them as an impediment to repentance. However, failure to recognise the traditional health system can result in dangerous situations, including toxic drug-herb interactions, a failure to administer the most effective treatments (Guan & Chen 2012) and cases of delayed treatment (Barker *et al.* 2006:670, 672) or even abandoned treatment (Amoaha *et al.* 2014:92).

At a meeting of the African Forum, on the Role of Traditional Medicine in Health Systems, one of the recommendations made by the World Health Organization was to establish mechanisms that would facilitate strong co-operation between traditional healers, scientists, and clinicians with acceptable arrangements for improved and loyal collaboration (World Health Organization 2002:1). The World Health Organization urged member states, of which South Africa is one, to prepare specific legislation to govern the practice of traditional medicine as part of the national health legislation (World Health Organization 2002:30). The promulgation of the *Traditional Health Practitioners Act*, No. 35 of 2004 (amended as Act 22 of 2007) by the South African government is the culmination of such efforts (*Traditional Health Practitioners Act* 22 of 2007).

In South Africa, it is estimated that 80% of South Africans consult traditional healers before consulting modern medicine (Latif 2010:1). As both types of health practitioners are working within the same communities, their respective practices may have had a synergistic or detrimental effect on the other’s practice to the benefit or disadvantage of the consumers of health services. Thus, collaboration of both systems is needed (Adams *et al.* 2009:793; Frenkel *et al.* 2008:178; Torri 2012:34). Currently, in South Africa, the only regulated working relationship or professional collaboration, between allopathic health practitioners and traditional surgeons, is regulated through the application of *Health Standards in Traditional Circumcision Act*, No. 66 of 2001 (Province of the Eastern Cape 2001). A study conducted by Gqaleni *et al.* (2011) on biomedical and traditional healing collaboration, on HIV and AIDS in KwaZulu-Natal, shows that collaboration between traditional and allopathic healthcare workers can benefit communities significantly. However, in the Eastern Cape Province, no studies could be found regarding the collaborative relationship between the two health systems. Therefore, there is no clear information as to whether or not, to what extent, or how the allopathic health practitioners were collaborating with the traditional health practitioners prior to the legalisation of traditional medicine in 2004.

The overall aim of the study was to explore and describe the collaborative professional relationship between allopathic and traditional health practitioners regarding the legalisation of traditional healing, and these health practitioners’ views regarding their collaborative relationship as role-players in the healthcare delivery landscape of the Amathole District in the Eastern Cape, South Africa.

## Research method and design

### Setting

The Amathole District is made up of five Local Service Areas or health sub-districts. Participants in this study were drawn from towns and their surrounding rural and informal settlement areas in this district. The study was conducted in the context of an environment characterised by racial and cultural diversity, within the ambit of a culture peculiar to the Xhosa ethnic group, and in the broader context of indigenous knowledge systems as practiced for decades by the black communities throughout the African continent.

### Design

The need to explore and describe the collaborative relationship between allopathic and traditional health practitioners, as role-players in the healthcare delivery system in the Amathole District, arises from a lack of documented information regarding this relationship. The approach used in this research study was, therefore, qualitative, exploratory, descriptive, and contextual in nature. A qualitative design was chosen using focus group interviews and unstructured individual interviews, as the viewpoints of allopathic and traditional health practitioners were captured in their own words and transcribed verbatim. An exploratory study aims at uncovering the relationships and dimensions of a phenomenon (Burns & Grove 2009:747).

### Participants

The target population comprised three groups of participants. Purposive sampling was used for Group One and Two, to consciously select certain participants to include in the study (Tashakkori & Teddlie 2003:713) based on their ability to best answer the research question (Creswell 2009:185). Snowball sampling was undertaken for Group Three using community members to find participants with specific traits and who are known and trusted in the community who might be difficult to identify by ordinary means (Polit & Beck 2013:517). The sample included people from rural and urban areas, a variety of different health practitioners, genders and racial groups.

Sampling was concluded when saturation of data were reached (Krueger & Casey 2000:26). Data saturation was reached for both the focus group interviews (Group One) and the unstructured individual interviews (Group Two and Three) when transcribing was drafted directly after each interview, and it became evident that no new data were forthcoming. One final focus group interview or individual interview was then held to conclude that all data had been captured.

A pilot study was undertaken by conducting an interview with one participant from each group. These participants met all the selection criteria, but were not included in the 28 participants who participated in the main study. The aim was to determine if the questions generated information that the researcher could use, and to establish if the interview technique was effective (Polit & Beck 2013:195). The interviews from the pilot study were transcribed and analysed in the planned manner to determine whether themes could be identified or not.

**TABLE 1:** Participant groups, sampling methods and inclusion criteria outlines the groups of participants, sampling and criteria.

Groups	Sampling	Criteria
**Group One**		
Allopathic health practitioners (registered nurses, medical practitioners, and pharmacists).	Purposive sampling	Willing to participate.
		A minimum of two years’ experience as: a medical practitioner, registered nurse or a pharmacist.
		Belonging to any other racial group that has provided healthcare services to the Xhosa communities.
		Have worked in a rural or urban public hospital, clinic or community health centre.
**Group Two**		
Traditional healers (diviners, herbalists, traditional surgeons, and birth attendants).	Purposive sampling	Willing to participate.
		A minimum of two years’ experience as: the particular speciality as a traditional healer.
		Belonging to the Xhosa ethnic group.
		Having practised at home.
**Group Three**		
Allopathic health practitioners (nurses and enrolled nurses and traditional healers).	Snowball sampling	Willing to participate.
		A minimum of two years’ experience as a nurse and a traditional healer.
		Belonging to the Xhosa ethnic group.
		Have worked either in a rural or urban public hospital, clinic or community health centre or having practised at home.

## Data collection

The data-collection method used for Group One were focus group interviews, as the researcher was looking for a range of ideas or feelings regarding the collaborative relationship between allopathic and traditional health practitioners, concerning the legalisation of traditional healing, as well as these practitioners’ views regarding their collaborative relationship as role-players in the healthcare delivery landscape. The researcher was also trying to understand differences in perspectives between the two groups of participants (Krueger & Casey 2000:46). As a result of existing shortages in health care staff, unstructured individual interviews were conducted with participants from Group Two and Group Three, because of the difficulty that might be experienced in bringing these health workers together at a central venue at the same time, as a result of shortages in health care staff. The interviews were tape-recorded to ensure that the participants’ responses were quoted verbatim or as closely as possible. Modified participant observation, field notes, and literature control were also used as data sources.

### Data analysis

The tape-recorded data was transcribed verbatim and the transcription utilised as the database for the study. The data were analysed using Tesch’s method as described by Creswell (2009:192) to identify themes and sub-themes.

### Trustworthiness

In order to ensure trustworthiness of the study the raw data were sent for analysis to an independent coder who is familiar with qualitative research (Creswell 2009:192). The independent coder was provided with a clean set of the transcripts and a copy of the research question, aim and objectives and a guideline of how the data were analysed. A meeting was arranged with the independent coder for a consensus discussion on the themes and sub-themes reached independently.

## Results

A total of *n* = 28 participants took part, and were divided into Three groups. Group One consisted of 10 (*n* = 10) participants: 8 registered nurses, 1 pharmacist, and 1 medical practitioner. Participants were mostly black people (*n* = 7) and white people (*n* = 2) with only one mixed race participant, and 9 (*n* = 9) of the participants were female. The participants’ work experience varied from 13 years to 41 years of working in a number of healthcare facilities, including different wards in district and regional hospitals and psychiatric clinics. Group Two consisted of 14 (*n* = 14) traditional healers, consisting of 4 diviners, 4 herbalists, 3 traditional surgeons, and 3 birth attendants. The participants were mostly male (*n* = 10), practicing in local urban, rural and township areas. The participants’ level of education varied from illiterate to being in possession of a Doctoral Degree. Lastly, Group Three consisted of 4 (*n* = 4) participants: 3 registered nurses, and 1 enrolled nurse, who are also traditional healers. All participants were female and black, with work experience as allopathic practitioners ranging from 13 to 31 years and as a traditional practitioners from 3 to 9 years.

The findings will be discussed per participant group. Details of participants, such as gender, race or occupation are not included in the quotations as participants indicated they preferred not to be identified.

### Group One: Allopathic health practitioners

Two main themes were identified which were suggested as crucial to the effective collaboration between traditional and allopathic health practitioners. Firstly, the negative attitude of allopathic health practitioners towards traditional health practitioners was identified and, secondly, the mutual understanding (in the form of changing attitudes and communication) between the two health systems, enabling them to collaborate and complement each.

### Theme One: The negative attitude of allopathic health practitioners towards traditional health practitioners

Almost all allopathic health practitioners who participated in this study stated that they had a generally negative attitude towards traditional health practitioners, and often warned patients against seeking the services of traditional health practitioners:

‘We do have a negative attitude and ask a person [*patient*]: “Why didn’t you come to hospital? Now, can you see how you look like? What do you want us to do now?”’

Participants highlighted the fact that the negative attitudes they had developed as allopathic health practitioners emanated from the following practices of traditional health practitioners:

- the unscientific methods used by the traditional health practitioners in treating patients
- interference of traditional healers with the efficacy of hospital treatment
- and delays by traditional healers in referring patients to hospital.

The sub-themes identified are outlined in the sections that follow.

### Sub-theme One: The unscientific method used by traditional health practitioners

Participants expressed concern regarding the unscientific methods of traditional medicine, such as non-use of handwashing, non-sterile equipment, and the lack of measured prescription of traditional medicine according to the age and weight of the patient. The following statements underscore these concerns:

‘The reason why we discourage them from seeing traditional healers is because those medicines of traditional healers are not sterile and they do not wash the hands.’

‘… and the person is taking the treatment for an indefinite time and that is dangerous to the client because sometimes it is damaging internal parts of the client.’

All participants reported encountering patients who presented them with complications after consulting traditional health practitioners. The complications included, amongst others, distended abdomen, diarrhoea, dehydration, damaged internal organs, poisoning and sores around the feet, arms and ribs.

### Sub-theme Two: Interference of traditional healers with the efficacy of hospital treatments

Participants stated that in some cases traditional health practitioners and the patients’ relatives interfered with the efficacy of hospital treatment by supplying the patients with traditional medicine from home. These could potentially cause changes in drug interaction, possible deterioration, and sometimes the death of the patient. This concern is illustrated as follows:

‘The relative will come and give the medicine […]. While the hospital treatment was about to be effective, the condition suddenly changes and we won’t know that there is another type of medicine that is being given secretly.’

### Sub-theme Three: Delays by traditional healers in referring patients to the hospital

Allopathic health practitioners were concerned that traditional health practitioners were keeping patients under their care for too long and only referred patients when the patient’s condition was at an advanced stage, which is outlined in the following statement:

‘… and she would state that the child had been ill for two to three weeks and that if you asked why she was only bringing the child now she would say “as Xhosas we had jumped this way and that way”.’

Delays in referral resulted in prolonged hospitalisation and made it difficult to implement certain diagnostic, surgical, and medical procedures.

### Theme Two: Mutual understanding was suggested as being crucial to the effective collaboration between traditional and allopathic health practitioners

Mutual understanding between allopathic and traditional health practitioners was viewed by allopathic health practitioners as central and crucial to effective collaboration. As one participant commented:

‘If we want collaboration, each has to understand the other side. Each group needs to understand what the other is capable of doing and limitations of each.’

To increase the acceptance of traditional health practitioners and a better understanding of their capabilities by the allopathic health practitioners, participants suggested *meetings* and *awareness campaigns* of traditional health practices that involve the following:

- **Training and development** of the traditional healers were suggested, to assist them in understanding health issues, correct clinical procedures and the health system, including the *Traditional Health Practitioners Act*. Likewise, allopathic health practitioners need to acquire a basic understanding of the traditional healing systems, including culture and traditional healing in the nursing and medical curricula.
- **Undertaking research:** Participants felt that there was a need to undertake research to understand the capabilities of traditional healers to successfully treat patients.
- **Collaboration between the relevant professional councils** was suggested to ensure registration of traditional health practitioners to avoid illegal referrals of patients by allopathic health practitioners to traditional healers.

### Group Two: Traditional health practitioners

Two main themes were identified: Firstly, traditional health practitioners’ experienced relationship with allopathic health practitioners’, which was characterised by a one-sided referral system. Secondly, the participants’ suggested possible areas of collaboration with allopathic health practitioners.

### Theme One: The participants’ experienced relationship with allopathic health practitioners, characterised by a one-sided referral system

Participants stated that their working relationship with allopathic health practitioners before publication of the *Traditional Health Practitioners Act* had been characterised by a one-sided referral system, with traditional health practitioners referring patients to allopathic health practitioners:

‘If only they can stop depriving us of patients. […] They [*doctors*] keep a person saying to him/her “come again for injection, or come again to fetch your treatment.” […] They don’t send them. This must not be one-sided.’

However, traditional health practitioners did refer patients to allopathic health practitioners as they had the knowledge, skills, technology, and equipment to investigate diseases and better manage the patients:

‘Personally I feel we must hand over to doctors, especially difficult labour. Doctors appear to have a good “hand” because they are educated. A doctor can see inside a person and see the position of a baby. They use instruments and see things that are inside.’

Another traditional health practitioner also referred patients depending on the cause of illness, as certain causes should rather be treated by traditional health practitioners:

‘A person is sick, because there are three things that make a person to be sick. […] It’s natural diseases, that’s the first one. There are many such diseases-things like measles, for instance. The second cause, she is sick, because of her “home things” (izinto zakowabo). Maybe she needs a “cultural necklace” (intambo) or a cultural ritual like “imbeleko” for enuresis in a person over 10 years. The third one is a “deliberate thing”, a man-made disease (yinto yangabom). You see now, doctors will not be able to treat your ‘home thing’ or refer you. Those instruments will not say this is a “deliberate thing” that this person is suffering from. But with natural diseases, the instruments will tell.’

### Theme Two: Participants suggested possible areas of collaboration with allopathic health practitioners

Participants generally worked in collaboration with allopathic health practitioners, but they clearly stated that the two systems should run parallel with identified areas of collaboration. As one participant stated:

‘This is a profession in its own right, that has to stand on its own.’

However, the participants suggested that areas of collaboration included *sharing of resources* such as the budget, health facilities like hospitals and clinics, equipment and information.

### Group Three: Nurses who are also traditional healers

Two themes emerged from this group. Firstly, role conflict related to their own professional role, expectations from colleagues and expectations from management when working in the clinical area whilst advocating for capacity building of traditional and allopathic health practitioners. Secondly, capacity building aimed to prepare the facilitation of collaboration and the cross-referral of patients, which will be outlined in the section which follows.

### Theme One: Participants experienced role conflict at different levels whilst working in the clinical area

The participants stated that they experienced role conflict during their work in the clinical situation. Role conflict was created by: their own professional role, expectations from colleagues as they viewed matters from a different perspective, and expectations from management:

‘Sometimes you could see that it could make you to be taken in a bad light at work. When I am being told things by the ‘voices’ on the roof and I say those things, people say I am a schizophrenic.’

Participants were not forthcoming when discussing how they wanted to professionally collaborate with allopathic health practitioners. This prompted the researcher to develop strategies for possible professional collaboration between the two groups, which is included in the discussion.

Theme Two: Participants experienced a need for capacity building of traditional and allopathic health practitioners to facilitate collaboration and cross-referral of patients.

Participants felt that there was a need to build the capacity of traditional and allopathic health practitioners in order to facilitate collaboration and cross-referral of patients. A statement from one of the participants reflects this viewpoint:

‘We must stop criticising each other. If the two sides can be work-shopped, sit together around the table and share ideas, it can be easy to collaborate and refer a patient. By the way we have different blessings.’

## Ethical considerations

Ethical approval was obtained by the Nelson Mandela Metropolitan University’s ethics committee, with ethical approval number: H2004-HEA-NUR-001. Furthermore, the ethical acceptability of the study was ensured throughout the research process. First and foremost the researcher has an obligation to respect the rights, needs, values and desires of the participants. Some of the measures that were taken to uphold ethical principles were to obtain written permission from each participant to conduct the study, articulate the research objectives verbally and in writing to make them clearly understood by the participants, including a description of how data would be used and how anonymity and confidentiality of participants would be ensured. Ethical clearance was obtained with the ethical research board from the relevant educational institution.

## Discussion

Both allopathic and traditional health practitioners experienced negative attitudes and practices. The allopathic health practitioners expressed a negative attitude towards the traditional health practitioners. One of the reasons mentioned was the unscientific methods used by traditional health practitioners in treating patients. Similar concerns were expressed in a multi-method study conducted by Peu (2000:95) on the attitude of community health nurses towards the integration of traditional health practitioners in primary healthcare in the North-West Province. Community health nurses raised concerns about the traditional healers’ unhygienic practices and felt that this was a constraint that could hinder the integration of allopathic and traditional healing systems (Peu 2000:95).

A second aspect contributing to the negative attitude towards the traditional health practitioners includes the interference of traditional healers with the efficacy of hospital treatments. In a study by Peu (2000), participants, specifically traditional healers, mentioned that they were also concerned that mixing traditional and western medicine could delay the healing process or nullify the treatment process and cause complications and, thus, warned their patients about it (Peu 2000:140).

A third factor, namely the traditional healers’ delays in referring patients to the hospital, also contributed to the negative attitude towards traditional health practitioners. Summerton (2006:21) also highlighted the tendency of traditional health practitioners to refer the patients to a western health facility as a last resort, when the patient was in the final stages of illness, with minimal chances of successful treatment interventions. Similarly a qualitative study conducted by Sorsdahl, Stein and Flisher (2010:591), on traditional healers’ attitudes and beliefs regarding referral of the mentally ill to western medical practitioners in South Africa, found that traditional healers’ referral to western care is considered a temporary measure, or a last resort, as they do not feel respected by allopathic health practitioners.

As negative attitudes were experienced towards each other by both parties, mutual understanding was suggested as crucial for effective collaboration between traditional and allopathic health practitioners. Peu (2000:140) concedes that a lack of appropriate knowledge and understanding by each party, about the other’s profession, is a constraint that could hinder the integration of traditional healers in primary healthcare. Similarly Madiba’s (2010:219) cross-sectional survey study to determine allopathic practitioners’ views on collaboration with traditional health practitioners in Botswana, also revealed that allopathic practitioners, besides using a one-way referral system, are not willing to collaborate. Furthermore, Torri’s (2012) case study in Chile regarding the applicability of equal acknowledgement of the traditional versus the allopathic health systems, found that a change of cultural viewpoints, mind-set, and mutual respect of different cultures is needed in order to create space for collaboration (Torri 2012:47).

In the light of this, the following strategies are suggested by the participants: Firstly, an investigation should be conducted to determine whether or not these negative attitudes are just misconceptions or reality. In addition, to limit misconceptions mutual understanding through communication should be established. Various ways of fostering mutual understanding have been reported by different studies. Meetings and training on health issues were recommended to foster mutual understanding and Mototo (1999:102) suggested holding regular meetings regarding health-related issues and basic health matters (Peu 2000:140), and training and development of traditional healers by nurses. A second strategy recommended was collaborative research between different types of health practitioners (Van Huyssteen 2007:172).

The traditional health practitioners’ negative attitudes to allopathic health practitioners was characterised by a one-sided referral system. Peu’s (2000:127) study confirmed this finding, revealing that 91.2% of the participants suggested the creation of an officially recognised referral system between traditional healers and community health nurses; the rationale being that both types of practitioners were consulting patients. However, mutual referral is only achievable in a climate where people respect one another’s uniqueness and competency. Mulaudzi (2001:19), therefore, suggests that healers from the respective backgrounds should have basic training regarding each other’s medical expertise, and that such mutual interchange would benefit both patients and practitioners.

In terms of collaboration between traditional and allopathic healers it is important to distinguish collaboration from other types of interactions, namely, shared goals, clear responsibilities, and mutual participation and use of resources (Mattessich 2005). Mattessich (2005) adds that the team members enter into the collaborative relationship with a specific purpose and objectives to be attained.

In this study, the traditional health practitioners highlighted areas of collaboration, such as the mutual use of resources (budget, physical health facilities, and equipment) and the sharing of information. In terms of budget sharing Kubukeli (1997:917) argues that because traditional healers treat about 80% of the population, they ought to share in the country’s healthcare budget. Nevertheless, Freeman and Motsei (1992:1188) expressed some reservations about accommodating an estimated 150 000 traditional healers in the country’s budget, and maintain that the health budget in South Africa is already stretched to the point where inadequate services are provided and the staff are underpaid.

The provision of physical health facilities for traditional healers is a subject that was raised as far back as 30 years ago. For instance, in a qualitative study conducted by Ojanuga (1981:410), medical practitioners who participated in the study suggested that traditional healers should have their own hospitals, and those running herbal healing homes should be given government subsidies for capital development, as these homes were often located in areas where there were no hospitals.

Regarding equipment, a qualitative study conducted in KwaZulu-Natal, by Mchunu and Bengu (2004:41), on the knowledge and attitudes of traditional birth attendants towards HIV and AIDS and their beliefs related to perinatal care, also revealed a need to provide traditional birth attendants with equipment like transport and delivery packs. The study also indicated a need to assist birth attendants with disinfection of their delivery equipment (Mchunu & Bengu 2004:49).

Challenges remain regarding the sharing of information pertaining to the patient, with Smart (2005:2) emphasising the need to clarify confidentiality issues first, before medical practitioners will feel free to share patient information with traditional healers.

A unique finding from this study arose from the nurses who are also traditional healers and who reported experiencing role conflict as a result of their own professional role, expectations from colleagues, and expectations from management whilst working in the clinical area. This group further suggested building the capacity of traditional and allopathic health practitioners in preparation for facilitating the collaboration and cross-referral of patients. No literature could be found that related to the role conflict of nurses who were also practising traditional healers. However, Mellish and Paton (2010:125–130) provide professional guidance regarding conflict between social behavioural norms and professional values and norms. Mellish and Paton (2010:128) further advise that in such a situation, professional norms should always be upheld, such as maintaining a professional image and ethical standards. Whilst the authors’ advice is acknowledged as being professionally sound, it may be interpreted as a dissonant chord with professionals wedged between their professional and personal values.

Lastly, emerging from this study is the need to address the challenges around capacity development to facilitate collaboration. Capacity building posed a challenge as most of the traditional healers were illiterate or poorly educated and would, thus, need to first undergo basic education and training. Steyn and Muller (2000:8), in exploring the possibility of incorporating traditional healers into the westernised medical efforts to combat cancer, highlighted the need for using pictures, illustrated pamphlets, magazines, and other material that was simple to understand and which would suit the level of education of a traditional healer.

### Practical implications and recommendations

Firstly, based on the findings in this study, it is recommended that as the traditional health system runs as a parallel system to the allopathic health system, there are shared areas of collaboration, namely, sharing resources such as budget, equipment, facilities, and information, as the two healing systems are premised on different ideological stances. The sharing of facilities does not imply that traditional health practitioners should be allowed to treat their patients whilst they are hospitalised, as their treatment modalities may differ from western methods and their traditional medicines could interact with medicines prescribed by the medical practitioners.

Secondly, similar to the referral policy developed in Amathole District, collaboration can be encouraged through formal policies. Therefore, the sharing of facilities refers to clinics which need to enhance collaboration between traditional and allopathic practitioners, to enable traditional health practitioners to freely refer their patients to these clinics. This can already be seen in the strategies to promote professional collaboration in the Amathole District, through the development of a referral policy. The referral chain outlined in this policy has to accommodate traditional health practitioners as they are the first contact made by African communities in search of health services, even before presenting themselves to clinics (Amathole Municipality District 2012:46).

Thirdly, practices of traditional healers that could be potentially disadvantageous to patients need to be rectified by capacity building, through the proposed training, regarding signs and symptoms of common diseases in this district which include tuberculosis, diabetes mellitus, hypertension, HIV and AIDS, sexually transmitted diseases (STDs), and children’s diseases.

Fourthly, it is also suggested that there is a representative for traditional health practitioners in all governance structures in the Province, especially for primary healthcare services. In this way, traditional health practitioners have the opportunity to interact with colleagues who are western-trained practitioners and gain knowledge and insight from them, and vice versa. Although the South African government acknowledged the need for such an integration of systems, establishing the Interim Council of Traditional Healers, the goal of integrating the Traditional Health Medicine into the national health system still has to occur (Ramokgopa 2013).

Fifthly, it is suggested, by the allopathic health practitioners, that policy-makers in the Department of Health consider the inclusion or absorption of traditional surgeons and traditional birth attendants into the provincial health system as community health workers. However, to be included, the traditional practitioners’ skills and knowledge should be enhanced through short courses and evaluations which are accredited by the relevant councils. At an educational level, it is suggested by the allopathic health practitioners that the Department consider using the nurses and medical practitioners, who are also traditional health practitioners, to provide in-service training for traditional health practitioners, as they understand both worlds, especially the intricacies of traditional healing. In-service training is needed to build bridges between the two system at early career stages and the culture of traditional healing and alternative healing systems should be included in the curriculum of nursing, medicine, and pharmacy to ensure that these students become acquainted with alternative types of healing at an early stage of their careers according to the allopathic health practitioners. Traditional health practitioners should also participate in all strategic planning workshops and strategic conversations hosted by the Department of Health. Such workshops and meetings will provide an opportunity for the two groups to address areas of concern, including any practices which undermine the practices of the other group. Workshops and meetings could also be used to openly discuss each other’s practices, acknowledging the limitations of each system and discussing how to use the strengths of each system to complement each other (UNAIDS 2006:14).

Finally, more research is needed regarding the attitudes of communities on the integration of the traditional healing system into the national health system. The views of different ethnic groups must be elicited to ensure a holistic trans-cultural perspective.

### Limitations of the study

The gazetting of the *Traditional Health Practitioners Act* 35 of 2004 (amended as Act 22 of 2007) – to regulate traditional healthcare services to ensure the efficacy, safety, and quality of those services – caused scepticism amongst one of the groups of participants (traditional health practitioners) with regard to the goal of this study. These participants suspected that providing a regulatory framework was a government ploy to invade their practice, or have them arrested for errors in their practice. Requesting them to sign the consent form compounded their suspicion and scepticism about the study, resulting in some participants, especially the birth attendants, being cautious and brief when responding to research questions (and not spontaneous and elaborative), and as such, much probing had to be undertaken to gain more information.

A second limitation was the use of only one focus group for the allopathic health practitioners, but the reality of staff shortages in the clinical situation in all health facilities in the Amathole District was a prohibiting factor. However, data which were derived from this focus group was rich and could be confirmed with the data of Group Three.

Lastly, the study was completed in the Eastern Cape Department of Health at the time when a new Service Transformation Plan was being introduced; coupled with the revision of the Service Delivery Model, including the de-complexing of hospital complexes and the de-clustering of district hospitals to their original status as separate entities as they were before introducing the concept of complexes and clusters. These structural changes may impact on the nomenclature that has been used in this study.

## Conclusion

In South Africa, the existence of context-specific issues and diseases such as Multiple and X-treme drug resistant Tuberculosis, HIV and AIDS, and lifestyle diseases, requires a concerted effort to address them. Facilitating collaboration between traditional and allopathic health practitioners is imperative considering the realities of staff shortages and the quadruple burden of diseases challenging South Africa. Suggestions were made regarding professional collaboration between both health systems in terms of change of attitude, communication and capacity building.
